# Single‐Dose, Intravenous, and Oral Pharmacokinetics of Isavuconazole in Dogs

**DOI:** 10.1111/jvp.13510

**Published:** 2025-04-08

**Authors:** Yishan Kuo, Zhong Li, Lauren E. Forsythe, Jennifer M. Reinhart

**Affiliations:** ^1^ The Department of Veterinary Clinical Medicine University of Illinois Urbana‐Champaign Urbana Illinois USA; ^2^ The Duke Proteomics and Metabolomics Core Facility Duke University Durham North Carolina USA

**Keywords:** antifungal, aspergillosis, dog, isavuconazole, pharmacokinetics, triazole

## Abstract

Isavuconazole, a triazole antifungal used in humans for invasive fungal infections, may be effective for treating canine fungal infections, although data on its use in dogs is limited. This study aimed to determine the pharmacokinetics and safety of a single dose of isavuconazole in dogs, administered both intravenously and orally. Six healthy dogs received 186 mg isavuconazonium sulfate in a crossover design, with blood samples collected over 28 days and an 8‐week washout period. Plasma isavuconazole and isavuconazonium concentrations were measured by liquid chromatography/mass spectrometry, and pharmacokinetic parameters were determined by non‐compartmental analysis. Isavuconazole was well tolerated, with key findings including intravenous clearance at 350 ± 112 mL/kg/h, volume of distribution at steady state at 9.8 ± 4.5 L/kg, and a terminal half‐life of 90 ± 44 h. For oral administration, the maximum concentration was 0.60 ± 0.27 μg/mL, time to maximum concentration was 6.73 ± 2.45 h, terminal half‐life was 125 ± 80 h, and the area under the curve was 7.44 ± 2.39 μg h/mL. Oral bioavailability was 81.4% ± 12.8%. These results suggest isavuconazole has a long half‐life in dogs and is well absorbed orally when administered in the fasted state. Further studies are warranted to establish a therapeutic regimen in dogs.

## 1 Introduction

Systemic mycoses are severe, life‐threatening diseases in dogs that can affect multiple body systems. Those caused by dimorphic fungi (e.g., blastomycosis, histoplasmosis) are most common and generally respond to first‐generation azole antifungal drugs including itraconazole and fluconazole. Survival rates for these infections range from about 60% to 90% (Mazepa et al. 2011; Wilson et al. 2018). Although the overall prognosis is fair, there is a subset of dogs that could benefit from additional treatment options. Invasive mold infections are rarer than infections with dimorphic fungi and carry a significantly poorer prognosis with survival as low as < 10% in one report (Elad 2019). Disseminated aspergillosis, the most common invasive mold infection in dogs, is intrinsically resistant to fluconazole and responds poorly to single‐agent itraconazole with a median survival time of only 63 days (Sykes 2013; Schultz et al. 2008; Elad 2019). Multi‐agent antifungal therapy and/or treatment with the second‐generation azole antifungal posaconazole may improve survival time in systemic aspergillosis, but most dogs eventually succumb to their disease (Corrigan et al. 2016; Lim et al. 2022). As further barriers to treatment, many currently used systemic antifungal agents have erratic oral bioavailability, particularly when administered without food, and inhibit their own metabolism by cytochrome P450 enzymes leading to nonlinear pharmacokinetics and unpredictable plasma drug concentrations (Boothe 2011; Lewis 2011). These factors highlight the need for improved therapies for canine deep mycoses, particularly invasive fungal infections.

Isavuconazole (Cresemba; Astellas Pharma US Inc., Northbrook, IL) is a second generation, triazole antifungal drug approved in 2015 for the treatment of invasive aspergillosis and mucormycosis (a Zygomycetes infection) in people (Cresemba 2022). Isavuconazole has in vitro activity against many fungal pathogens important in veterinary medicine including *Blastomyces*, *Coccidioides*, *Cryptococcus*, *Aspergillus*, and multiple non‐*Aspergillus* mold species and has demonstrated efficacy against fluconazole‐resistant strains of *Histoplasma* and *Cryptococcus* spp. (González 2009; Guinea et al. 2008; Spec et al. 2018; Thompson et al. 2009). Isavuconazole is supplied as isavuconazonium sulfate, a water‐soluble prodrug that is quickly cleaved to the active form of isavuconazole by plasma esterases (Schmitt‐Hoffmann et al. 2006). It is available as oral and injectable formulations. In humans, isavuconazole has high oral bioavailability (~98%) and absorption is not affected by food intake or gastric pH (Schmitt‐Hoffmann et al. 2016). It is cleared by the liver but does not appear to induce or inhibit the cytochrome P450 enzymes, which leads to linear pharmacokinetics and predictable drug concentrations. Isavuconazole is highly protein bound (~98%) and has a long half‐life of approximately 130 h in people (Schmitt‐Hoffmann et al. 2006). Pharmacokinetic study of isavuconazole in cats revealed similar results with high protein binding, long half‐life, and high oral bioavailability (Woerde et al. 2022). A recent pharmacokinetic study of isavuconazole in Beagles revealed rapid plasma conversion and relatively short half‐life at 10–15 h, regardless of the route of administration (McQuinn et al. 2024).

Isavuconazole has many demonstrated benefits in people, including broad spectrum antifungal efficacy, predictable pharmacokinetics, and high oral bioavailability. It is possible that isavuconazole could have similar benefits in the treatment of invasive fungal infections in dogs. Therefore, the object of this study is to establish the single‐dose pharmacokinetic profiles of isavuconazole intravenous and oral isavuconazonium sulfate in healthy dogs. We hypothesize that, similar to humans and cats, isavuconazole in dogs will be well absorbed, and have a long half‐life with minimal adverse effects. This represents a first step in generating recommendations for the rational use of isavuconazole in canine invasive fungal infections.

## 2 Material and Methods

### 2.1 Study Population

Six dogs were recruited from the pet population of individuals affiliated with the University of Illinois College of Veterinary Medicine, with informed owner consent. To be included, dogs had to be at least 1 year of age, weigh at least 20 kg, and be systemically healthy. All dogs were determined to be healthy based on a complete physical examination performed by a board‐certified small animal veterinary internist (JR), complete blood cell count, serum biochemistry, and urinalysis during an initial screening visit 1–2 weeks prior to drug administration and sampling. Dogs were excluded if they had chronic illness, clinically relevant abnormal laboratory results, or were receiving any medications other than monthly heartworm and flea/tick preventatives. This study received approval from the University of Illinois Institutional Animal Care and Use Committee (protocol #22065).

### 2.2 Experimental Design

This study was performed in two phases: an intravenous phase and an oral phase. Three dogs underwent serial plasma drug monitoring following a single intravenous dose of 186 mg isavuconazonium sulfate (Cresemba for injection, 100 mg isavuconazole equivalent, Astellas Pharma US Inc., IL). Subsequently, after at least an 8‐week washout period, the same dogs underwent serial plasma drug monitoring following a 186 mg oral dose of isavuconazonium sulfate (Cresemba Capsules, 100 mg isavuconazole equivalent, Astellas Pharma US Inc., IL) on an empty stomach. The other three dogs received the oral product first, followed by the washout period, and then the intravenous product. The 8‐week washout period was based on a 130‐h half‐life reported in humans and a recommended a washout period of at least 10 times the half‐life between treatments for pharmacokinetic studies (130 h × 10/24 h/day = 54 days, approximately 8 weeks) (Toutain and Koritz 1997).

### 2.3 Drug Administration and Sample Collection

All dogs were fasted for at least 8 h prior to hospital admission. Dogs were then sedated with dexmedetomidine (Dexdomitor, Zoetis Inc., Parsippany‐Troy Hills, NJ) at 2–4 μg/kg and butorphanol (Torbugesic‐SA, Zoetis Inc.) at 0.2 mg/kg intravenously for the placement of a sterile jugular venous catheter (MILA International Inc., Florence, KY) using the modified Seldinger technique. For the intravenous phase, an intravenous catheter was placed in a cephalic vein for the administration of isavuconazonium sulfate at a separate site from sampling. A reversal agent, atipamezole (Antisedan, Zoetis Inc.), was administered intramuscularly at 0.02–0.04 mg/kg, and dogs were monitored during recovery. Following recovery, the dogs were fed a meal of their normal diet and underwent a second fast for at least 12 h overnight before drug administration (intravenous and oral phases) and sampling the next morning, with free access to water. For the intravenous phase, dogs were administered 186 mg isavuconazonium sulfate for injection as an infusion over 1 h in 125 mL of 0.9% sodium chloride, after which the cephalic catheters were removed. For the oral phase, dogs were administered a single 186 mg isavuconazonium sulfate capsule in a small meatball of wet food.

In both phases, blood samples (5 mL) were collected from the sampling catheter using the three syringe technique before drug administration (day 0, time 0 h) and at 0.25, 0.5, 0.75, 1, 2, 4, 6, 8, 12, and 24 h (day 1) from the start of infusion or oral administration. The sampling catheters were then removed, and dogs were discharged to their owners following the 24‐h sample collection. Owners were asked to return their dogs on days 2, 3, 6, 8, 10, 13, 17, 22, and 28 of the study periods for sample collection via jugular, cephalic, or saphenous venipuncture (Schmitt‐Hoffmann et al. 2006). This sampling duration was selected to ensure that at least 80% of the total area under the curve would be captured by the data in this study (Toutain and Koritz 1997). On occasion, owners were allowed to shift follow‐up visits by a day to better accommodate their schedules; the actual time of collection was used in the pharmacokinetic analysis. Following each collection, blood was placed into EDTA‐containing tubes; 10 μL of 2 M citric acid and 10 μL of 0.1 M paraoxon were added to each sample immediately to avoid degradation of isavuconazonium (Schmitt‐Hoffmann et al. 2006) and refrigerated. Plasma was separated by centrifugation at 1500 *g* at 4°C within 4 h of collection and stored at −80°C until analysis.

### 2.4 Plasma Isavuconazonium and Isavuconazole Quantification

Plasma isavuconazonium and isavuconazole concentrations were quantified at the Duke Proteomics and Metabolomics Core Facility. Samples were analyzed using liquid chromatography/tandem mass spectrometry (LC/MS/MS). Samples (30 μL) were spiked with 50 μL d4‐isavuconazole internal standard (200 ng/mL) and 30 μL acetonitrile. These were vortexed for 1 min, kept at −20°C for 20 min, centrifuged at 20,000 *g* for 5 min, transferred to a 96‐well plate, and centrifuged again at 1500 *g* for 2 min prior to loading onto the autosampler. Calibration standards and quality controls were prepared by serial dilution in acetonitrile with blank dog plasma as the matrix. For isavuconazole, calibration standard concentrations were 20, 10, 4, 1, 0.2, 0.04, 0.01, 0.002, and 0.0004 μg/mL, and the low, medium, and high quality control concentrations were 0.05, 0.2, and 1 μg/mL. For isavuconazonium, calibration standard concentrations were 2.5, 1.25, 0.5, 0.125, 0.025, 0.005, 0.00125, and 0.00025 μg/mL, and the low, medium, and high quality control concentrations were 0.02, 0.08, and 0.4 μg/mL.

Samples were analyzed with the Sciex QTrap 6500+ system (Framingham, MA) with Waters Acquity I‐class plus UPLC. Software Analyst 1.7.3 was used for data acquisition. Separation was performed on a Waters Acquity (Milford, MA) UPLC BEH C18 column (2.1 × 50 mm, 1.7 μm) with mobile phase A (0.1% formic acid in water) and mobile phase B (0.1% formic acid in acetonitrile). The flow rate was 0.5 mL/min unless noted otherwise. The linear gradient was as follows: 0–0.5 min, 95% A; 1.25 min, 75% A; 1.7 min, 50% A; 2.2 min, 30% A; 2.7–2.95 min, 0% A; 3–4.6 min, 0% A (0.85 mL/min); 4.65–5.5 min, 95% A. The autosampler was set at 10°C and the column was kept at 45°C. The injection volume was 5 μL. Retention times were 2.6 min for isavuconazole and 2.0 min for isavuconazonium.

Mass spectra were acquired under positive electrospray ionization (ESI) with the ion spray voltage of 4000 V. The source temperature was 450°C. The curtain gas, ion source gas 1, and ion source gas 2 were 35, 55, and 65 psi, respectively. Multiple reaction monitoring (MRM) was used for quantitation of isavuconazole (m/z 438.1 → m/z 127.0), isavuconazonium (m/z 717.2 → m/z 236.1), and d4‐isavuconazole (m/z 442.1 → m/z 373.1) as the internal standard for both analytes.

All data was analyzed in software Skyline (version 23.1.0.268). It includes raw data import, peak integration, and a regression fit with 1/× weighting for the calibration curves. All curve fits had a correlation coefficient > 0.99. The lower limits of quantification (LOQ) were 0.0004 and 0.00025 μg/mL for isavuconazole and isavuconazonium, respectively, and were defined 10× the signal‐to‐noise ratios of the calibration curves. The recovery, accuracy, precision, and stability for each analyte are included in Tables S1–S3.

### 2.5 Pharmacokinetic Analysis

Continuous data are presented as geometric mean ± standard deviation. Pharmacokinetic analysis for plasma isavuconazonium and isavuconazole administered intravenously and orally in dogs were performed in Phoenix WinNonLin (Cetara USA Inc., Princeton, NJ) using non‐compartmental methods. Calculated parameters included: maximum plasma concentration (*C*_max_), time at *C*_max_ (*T*_max_), terminal rate constant (*λ*_z_), terminal half‐life (*t*_1/2_), observed area under the curve (AUC_0–*t*_), area under the curve extrapolated to infinity (AUC_0–∞_), percent of the AUC_0–∞_ extrapolated to infinity (AUC_%extrap_), observed area under the moment curve (AUMC_0–*t*_), area under the moment curve extrapolated to infinity (AUMC_0–∞_), percent of the AUMC_0–∞_ extrapolated to infinity (AUMC_%extrap_), and mean residence time (MRT). Clearance (Cl), volume of distribution at steady state (*V*_ss_), and volume of distribution by the area method (*V*_z_) were calculated for the intravenous phase, and bioavailability (*F*) was calculated for the oral phase. AUC_0–*t*_ was calculated by the linear‐log trapezoidal method. At least three sampling points were used for the calculation of *λ*_z_ for each time‐concentration curve and were automatically selected by the software to optimize the fit of a mono‐exponential equation to the terminal portion of the curve. The effect of treatment order on pivotal parameters (*C*_max_, AUC_0–*t*_, AUC_0–∞_) for each analyte was assessed using a multivariate mixed model with route of administration and treatment order included as fixed effects and individual dog included as a random effect. This analysis was performed using SAS OnDemand for Academics (proc MIXED).

### 2.6 Adverse Events Monitoring

Dogs were monitored by research personnel during hospitalization and by owners after discharge using a standardized form. Specifically, dogs were monitored for the following adverse events: infusion site reaction, abdominal pain, lethargy/fatigue, rash, inappetence/anorexia, diarrhea, and vomiting. These events were graded using a scale (Table S4) modified from the Veterinary Cooperative Oncology Group‐Common Terminology Criteria for Adverse Effects (LeBlanc et al. 2021).

## 3 Results

### 3.1 Study Population

Six healthy dogs were enrolled in this study, with a mean age of 5.8 years (range 1–7 years) and a mean weight of 31.8 ± 8.2 kg (range 22.8–44.6 kg). The study population included three spayed females and three neutered males. The breeds included three mixed‐breed dogs and one of each of the following: Borzoi, Rhodesian Ridgeback, and Old English Sheepdog. The mean dose of isavuconazonium sulfate administered in each phase was 6.11 ± 1.39 mg/kg (range 4.25–7.95 mg/kg), which is equivalent to isavuconazole at 3.28 ± 0.53 mg/kg (range 2.28–4.27 mg/kg).

### 3.2 Intravenous Pharmacokinetics

**FIGURE 1:**
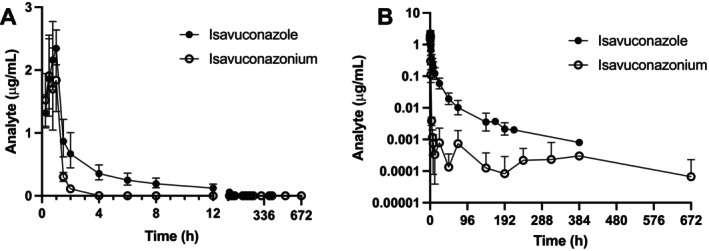
Time‐concentration curves of isavuconazole (black dots) and its prodrug, isavuconazonium (open dots) following intravenous administration of isavuconazonium sulfate to dogs. Data are presented on (A) linear and (B) semi‐log plots for ease of visualization. Note the first 12‐h period post‐administration has been expanded on the *x*‐axis in panel A. Some datapoints are not represented in panel B because values of 0 cannot be plotted on a semi‐log graph.

Pharmacokinetic parameters for the prodrug, isavuconazonium, and isavuconazole after a single intravenous infusion of isavuconazonium sulfate over 1 h are presented in Table 1. Plasma drug concentrations and pharmacokinetics for individual dogs are presented in Tables S5–S8. The plasma concentration‐time profiles of isavuconazonium and isavuconazole after intravenous administration of isavuconazonium sulfate are presented in Figure 1. Peak plasma concentrations of isavuconazonium and isavuconazole occurred during the infusion at 0.67 ± 0.25 h (range 0.5–1) and 0.85 ± 0.21 h (range 0.5–1), respectively, with isavuconazole having a higher *C*_max_ (2.60 ± 0.49 μg/mL) than isavuconazonium (1.92 ± 0.62 μg/mL). Following cessation of the infusion, isavuconazonium was rapidly eliminated (*t*_1/2_ 1.77 ± 1.59 h), while the *t*_1/2_ for isavuconazole was much longer at 90 ± 44 h. This resulted in a relatively low Cl (0.35 ± 0.11 L/kg/h) and large *V*_ss_ (9.8 ± 4.5 L/kg) for isavuconazole.

**TABLE 1:** Isavuconazonium and isavuconazole pharmacokinetic parameters for dogs administered intravenous isavuconazonium sulfate. Parameters are presented as geometric mean, standard deviation (SD), minimum, and maximum.

Parameter	Unit	Isavuconazonium				Isavuconazole			
		Mean	SD	Min	Max	Mean	SD	Min	Max
Dose	mg/kg	5.96	1.49	4.25	7.95	3.20	0.80	2.28	4.27
*λ*_z_	h^−1^	0.392	0.702	0.177	1.798	0.008	0.005	0.005	0.017
*t*_1/2_	h	1.77	1.59	0.39	3.91	90	44	41	147
*T*_max_	h	0.67	0.25	0.50	1.00	0.85	0.21	0.50	1.00
*C*_max_	μg/mL	1.92	0.62	1.25	3.04	2.60	0.49	2.06	3.25
AUC_0–*t*_	μg h/mL	2.23	0.52	1.80	3.02	9.04	2.34	5.48	11.47
AUC_0–∞_	μg h/mL	2.23	0.52	1.80	3.02	9.14	2.32	5.62	11.62
AUC_%extrap_	%	0.1	0.1	0.0	0.2	0.9	0.7	0.3	2.4
*V*_z_	L/kg	6.8	8.0	1.1	20.8	45.7	34.0	21.2	108.7
Cl	mL/kg/h	2671	952	1408	4408	350	112	213	559
AUMC_0–*t*_	μg h^2^/mL	1.89	0.30	1.55	2.27	208	102	109	341
AUMC_0–∞_	μg h^2^/mL	1.93	0.29	1.57	2.28	260	121	134	443
AUMC_%extrap_	%	1.0	1.3	0.1	3.6	15.8	11.8	4.9	38.8
MRT	h	0.36	0.10	0.25	0.50	28	9	16	38
*V*_ss_	L/kg	1.0	6.3	3.6	2.2	9.8	4.5	5.6	17.4

Abbreviations: AUC_%extrap_, percent area under the curve extrapolated; AUC_0–∞_, area under the curve extrapolated to infinity; AUC_0–∞_/*D*, area under the curve extrapolated to infinity normalized to dose; AUC_0–*t*_, observed area under the curve; AUC_0–*t*_/*D*, observed area under the curve normalized to dose; AUMC_%extrap_, percent area under the moment curve extrapolated; AUMC_0–∞_, area under the moment curve extrapolated to infinity; AUMC_0–*t*_, observed area under the moment curve; Cl, clearance; *C*_max_, maximum concentration; *C*_max_/*D*, maximum concentration normalized to dose; MRT, mean residence time; *t*_1/2_, terminal half‐life.; *T*_max_, time at maximum concentration; *V*_ss_, volume of distribution at steady state; *V*_z_, volume of distribution by the area method; *λ*_z_, terminal rate constant.

### 3.3 Oral Pharmacokinetics

**FIGURE 2:**
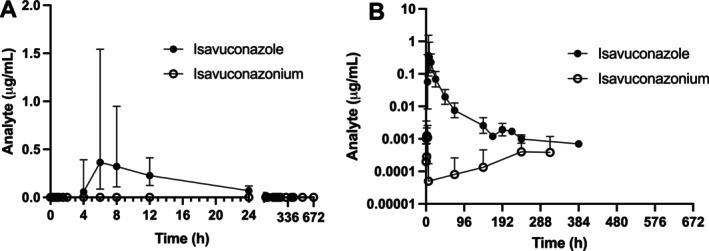
Time‐concentration curves of isavuconazole (black dots) and its prodrug, isavuconazonium (open dogs) following oral administration of isavuconazonium sulfate to dogs. Data are presented on (A) linear and (B) semi‐log plots for ease of visualization. Note the first 24‐h period post‐administration has been expanded on the *x*‐axis in panel A. Some datapoints are not represented in panel B because values of 0 cannot be plotted on a semi‐log graph.

Plasma isavuconazonium concentrations following a single oral dose of isavuconazonium sulfate were very low to undetectable for all dogs throughout the sampling period (Table S9) and so pharmacokinetic analysis was not performed. Pharmacokinetic parameters for plasma isavuconazole are presented in Table 2. Plasma drug concentrations and pharmacokinetics for individual dogs are presented in Tables S10–S11. The plasma isavuconazole concentration‐time profiles are presented in Figure 2. Maximum plasma concentrations of isavuconazole were reached at 6.73 ± 2.45 h after drug administration and were lower than those following intravenous administration (0.60 ± 0.27 μg/mL). Oral bioavailability of isavuconazole administered as isavuconazonium sulfate was 81.4% ± 12.8% in the fasted state. Treatment order did not significantly influence pivotal pharmacokinetic parameters for either route or analyte.

**TABLE 2:** Isavuconazole pharmacokinetic parameters for dogs administered oral isavuconazonium sulfate. Parameters are presented as geometric mean, standard deviation (SD), minimum, and maximum.

Parameter	Units	Mean	SD	Min	Max
Dose	mg/kg	3.20	0.80	2.28	4.27
*λ*_z_	h^−1^	0.006	0.003	0.002	0.011
*t*_1/2_	h	125	80	62	293
*T*_max_	h	6.73	2.45	6.00	12.00
*C*_max_	μg/mL	0.60	0.27	0.37	1.00
AUC_0–*t*_	μg h/mL	7.32	2.38	3.77	11.10
AUC_0–∞_	μg h/mL	7.44	2.39	3.85	11.25
AUC_%extrap_	%	1.5	1.0	0.8	3.5
AUMC_0–*t*_	μg h^2^/mL	207	96	98	365
AUMC_0–∞_	μg h^2^/mL	276	149	136	526
AUMC_%extrap_	%	21.7	12.3	12.4	45.9
MRT	h	37	19	21	73
*F*	%	81.4	12.8	68.5	98.2

Abbreviations: AUC_%extrap_, percent area under the curve extrapolated; AUC_0–∞_, area under the curve extrapolated to infinity; AUC_0–∞_/*D*, area under the curve extrapolated to infinity normalized to dose; AUC_0–*t*_, observed area under the curve; AUMC_%extrap_, percent area under the moment curve extrapolated; AUMC_0–∞_, area under the moment curve extrapolated to infinity; AUMC_0–*t*_, observed area under the moment curve; *C*_max_, maximum concentration; *F*, bioavailability; MRT, mean residence time; *t*_1/2_, terminal half‐life; *T*_max_, time at maximum concentration; *λ*_z_, terminal rate constant.

### 3.4 Adverse Events

Single doses of isavuconazonium sulfate were well tolerated when administered intravenously and orally, and no severe adverse events occurred during the study period. One dog experienced minor erythema at the peripheral catheter site where the intravenous drug was infused (VCOG grade 2). However, the lesion was superficial, and it was suspected to be due to dermal irritation from clipping of the hair compounded with self‐trauma rather than a true injection site reaction. No other adverse reactions occurred.

## 4 Discussion

This study was designed to evaluate the single‐dose intravenous and oral pharmacokinetics and safety of isavuconazole in healthy dogs. Our results indicate that, in dogs, isavuconazole has a large volume of distribution, low systemic clearance, prolonged half‐life, and favorable oral bioavailability. After intravenous administration, peak concentrations of isavuconazonium and isavuconazole were observed during or at the end of the infusion (range 0.5–1 h for both analytes) and the half‐life of isavuconazonium was relatively short (1.77 ± 1.59 h), compared to that of isavuconazole (90 ± 44 h). These findings indicate rapid conversion of the isavuconazonium into active isavuconazole in dogs. After oral administration, isavuconazonium concentration was very low to undetectable in all dogs at all timepoints. This also likely reflects rapid prodrug activation during a relatively slow oral absorption period (*T*_max_ 6.73 ± 2.45 h). The observed nonlinear kinetics of isavuconazonium may be attributable to the exceptionally LOQ in the assay. This raises the possibility that the detected variability reflects assay noise rather than true pharmacokinetic behavior.

Therapeutic plasma drug targets and optimal serum concentrations for isavuconazole have not been defined for canine deep mycoses. In one study, the modal minimum inhibitory concentrations (MICs) for isavuconazole against clinical *Aspergillus* spp. isolates were 0.5 μg/mL, but values ranged from 0.06 to > 16 μg/mL (Pfaller et al. 2018). Definitive pharmacodynamic targets have not been established for isavuconazole, but for other azoles, an AUC/MIC ratio appears to be the best predictor of treatment success (Lepak and Andes 2014). Canine‐specific isolate susceptibility to isavuconazole has not been performed and would be required prior to establishing a similar therapeutic target. In humans, serum isavuconazole trough concentrations > 1 μg/mL have been utilized in therapeutic drug monitoring to ensure efficacy (Andes et al. 2018). Isavuconazole concentrations at 24 h were well below this target in our study following both oral and intravenous administration; however, with the long half‐life detected, we expect significant accumulation in dogs and so steady‐state concentrations would likely be much higher.

The volumes of distribution for isavuconazole in dogs were quite large (*V*_ss_ 9.8 ± 4.5 L/kg, *V*_z_ 35.7 ± 34.0 L/kg) indicating wide tissue distribution and/or high protein binding. Although not directly assessed in our study, previous studies have demonstrated significant protein binding of isavuconazole in humans (98%) and cats (99%), as well as high protein binding of other azole antifungals in dogs (Bellmann and Smuszkiewicz 2017; Kendall and Papich 2015). The large volume of distribution combined with low systemic clearance (0.35 ± 0.11 L/kg/h) confers the prolonged terminal half‐lives of isavuconazole in dogs of 90 ± 44 h and 125 ± 80 h for intravenous and oral administration, respectively. These pharmacokinetic properties are similar to the observations from human and feline studies. In the recently published canine isavuconazole pharmacokinetic study (McQuinn et al. 2024), shorter half‐lives of 10 and 14.9 h were observed following intravenous and oral administration, respectively. A possible explanation is the lower LOQ in our assay (0.4 ng/mL vs. 20 ng/mL), which allowed detection of very small concentrations during the terminal phase. Based on our findings, the long terminal half‐life might allow infrequent isavuconazole dosing. In people, isavuconazole is dosed once daily (Cresemba 2022) and every other day administration has been suggested for cats (Woerde et al. 2022). The dose at which the drug should be administered depends on the target plasma concentrations for canine deep mycoses and establishing dose linearity for isavuconazole in dogs, both of which require further study.

The oral bioavailability of isavuconazole was 81.4% ± 12.8% ranging between 68.5% and 98.2% among the six dogs in this study. This is somewhat lower than the 98% bioavailability reported for people. Although the presence of food and gastric pH minimally impact isavuconazole bioavailability in humans, the same may not be true for dogs. As this was a first‐in‐species study, we elected to withhold food prior to drug administration to eliminate its effect on the pharmacokinetic profile. However, the impact of feeding warrants further investigation in dogs. The estimated oral bioavailability of isavuconazole is 88% in cats (Woerde et al. 2022). Animals were fed ad libitum in that study, so it is possible that administering isavuconazole with food may improve bioavailability in dogs as it does for other azole antifungal drugs. Aside from a possible mild injection site reaction, single doses of the intravenous and oral formulations of isavuconazole were well tolerated in dogs in our study. With its long half‐life, isavuconazole will accumulate during long‐term administration, so additional dose‐dependent adverse drug reactions are possible.

The major limitation of this study is its relatively small sample size of six subjects. Future research endeavors should aim to expand upon the findings presented here, establishing dosing regimens to achieve target steady‐state concentrations through multi‐dose studies. Additionally, interactions between isavuconazole and common veterinary drugs should be evaluated, as has been demonstrated for erdafitinib (Ruan et al. 2021). Further studies are warranted to assess repeatability in larger sample sizes, evaluate the impact of feeding on oral bioavailability, conduct pharmacodynamic assessments, and perform clinical trials to evaluate treatment efficacy and the incidence of adverse effects in the target population.

## 5 Conclusion

Isavuconazole may be a valuable addition to the current treatment options of invasive fungal infection in canine medicine. It demonstrated a long terminal half‐life and favorable oral bioavailability. Additional studies are required to establish a therapeutic regimen based on this report.

## Author Contributions

Y.K.: study execution, writing – original draft, writing – review and editing. Z.L.: methodology, study execution, formal analysis, writing – review and editing. L.E.F.: conceptualization, writing – review and editing. J.M.R.: conceptualization, methodology, study execution, formal analysis, writing – review and editing.

## Ethics Statement

The authors confirm that the ethical policies of the journal, as noted on the journal's author guidelines page, have been adhered to and the appropriate ethical review committee approval has been received. The authors confirm that they have adhered to either US or European standards for the protection of animals used for scientific purposes.

## Conflicts of Interest

The authors declare no conflicts of interest.

## Supporting information


Data S1

## Data Availability

The data that supports the findings of this study are available in the Supporting Information of this article.
