# Supplementary material for: Single‐Dose, Intravenous, and Oral Pharmacokinetics of Isavuconazole in Dogs
Source: J Vet Pharmacol Ther. 2025 Apr 8;48(4):234–40. doi: 10.1111/jvp.13510 (PMC12257257; doi:10.1111/jvp.13510)
Supplement: Supplementary file 1 — Data S1 [file JVP-48-234-s001.docx]

**Supplemental Data for “Single Dose, Intravenous and Oral Pharmacokinetics of Isavuconazole in Dogs”**

**Table S1** – Recovery analysis for LC/MS assay of isavuconazole and isavuconazonium in dog plasma.

|  | **Recovery (n=5)** | | |
| --- | --- | --- | --- |
| **isavuconazole (ng/mL)** | **50** | **200** | **1000** |
|  | 109.4 ± 2.9 | 99.5 ± 5.2 | 99.7 ± 1.4 |
| **isavuconazonium (ng/mL)** | **20** | **80** | **400** |
|  | 132.7 ± 3.4 | 98.1 ± 7.0 | 87.5 ± 2.6 |
| **d4-isavuconazole** | - 1. ± 4.3 | | |

Notes: (A) The listed values are mean recovery (%) ± SD (standard deviation).

(B) Recovery is calculated by the signal ratio of spiked standard in acetonitrile to spiked standard into blank plasma.

**Table S2** – Accuracy and precision analysis for LC/MS assay of isavuconazole and isavuconazonium in dog plasma.

|  |  | **within-run accuracy (n=5)** | | | **between-run accuracy (n=15)** | **Within-run precision (n=15)** | **between-run precision (n=15)** |
| --- | --- | --- | --- | --- | --- | --- | --- |
|  |  | **(Mean ± SD)** | | |  |  |  |
|  | **(ng/mL)** | **Day 1** | **Day 2** | **Day 3** |  |  |  |
| **isavuconazole** | **50** | 50.8 ± 0.9 | 49.8 ± 1.4 | 50.2 ± 1.3 | 50.3 ± 1.2 | 2.4 | 8.8 |
|  | **200** | 204.3 ± 5.3 | 201.5 ± 7.6 | 200.5 ± 5.9 | 202.1 ± 6.1 | 3.1 | 2.2 |
|  | **1000** | 1048.2 ± 23.1 | 1040.3 ± 28.4 | 1032.4 ± 19.1 | 1040.3 ± 23.1 | 2.3 | 1.5 |
| **isavuconazonium** | **20** | 18.4 ± 0.7 | 19.7 ± 0.3 | 18.5 ± 0.3 | 18.9 ± 0.8 | 2.9 | 7.3 |
|  | **80** | 77.3 ± 1.2 | 85.5 ± 2.3 | 81.2 ± 3.6 | 81.6 ± 4.2 | 3.1 | 10.8 |
|  | **400** | 399.6 ± 5.9 | 416.1 ± 5.1 | 411.8 ± 4.7 | 409.2 ± 8.7 | 1.3 | 4.5 |

**Table S3** – Stability analysis for LC/MS assay of isavuconazole and isavuconazonium in dog plasma.

|  |  | **Stability (n=5)** |  |
| --- | --- | --- | --- |
| **isavuconazole (ng/mL)** | **50** | **200** | **1000** |
|  | 48.2 ± 0.7 | 194.7 ± 2.9 | 969.6 ± 10.6 |
| **isavuconazonium (ng/mL)** | **20** | **80** | **400** |
|  | 19.4 ± 1.0 | 84.6 ± 5.3 | 428.8 ± 7.1 |

Notes: (A) The listed values are measured values ± SD.

(B) The samples are measured after 48 hours at 10°C.

**Table S4** – Adverse event monitoring scale as provided to study participant owners. This scale is modified from the Veterinary Cooperative Oncology Group-Common Terminology Criteria for Adverse Effects (LeBlanc et al., 2021).

| **Adverse Effect** | **Grade 0** | **Grade 1** | **Grade 2** | **Grade 3** |
| --- | --- | --- | --- | --- |
| Infusion Site Reaction | None | Mild swelling without pain or redness | Redness and/or pain with or without swelling* | Open sores* |
| Abdominal Pain | None | Mild pain, not limiting normal daily activities* | Moderate pain, somewhat limiting daily activities* | Severe pain, limiting daily activities^&^ |
| Lethargy/fatigue | Normal activity | Mild lethargy compared to normal, but able to perform normal daily functions with ease | Moderate lethargy compared to normal causing some difficulty performing normal daily functions* | Severe lethargy, difficulty ambulating^&^ |
| Rash | None | Bumps or redness covering < 10% of the body surface* | Bumps or redness covering 10-30% of the body surface* | Bumps or redness covering > 30% of the body surface* |
| Inappetence/anorexia | Normal appetite | Decreased interest in 1 meal | Decreased interest in all daily meals or complete anorexia for 1 meal* | Complete anorexia* |
| Diarrhea | Normal stools | Single episode of watery, unformed stools | Multiple episodes of watery, unformed stools in a day | Profuse, large volume watery stools; large volume blood in stools; dark/tarry stools; diarrhea that interferes with normal daily activity* |
| Vomiting | None | 1-2 episodes of vomiting, self-limiting, do not interfere with normal activity | > 2 episodes of vomiting, normal daily activity* | Profuse vomiting and/or vomiting that interferes with normal daily activity^&^ |
| Other | None | Please record any other adverse effects. Please contact study personnel with any concerns. | | |

* Please contact study personnel

^&^ Please take your pet to the University of Illinois Emergency Service and contact study personnel

If any adverse effect persists for > 48 h or if you have any concerns, please contact study personnel

**Table S5** – Isavuconazonium concentrations for dogs administered isavuconazonium sulfate intravenously. Dogs 1-1, 1-2, and 1-3 received the intravenous formulation first, while dogs 2-1, 2-2, and 2-3 received the intravenous formulation following the washout period.

| **Time (h)** | **Isavuconazonium (μg/mL)** | | | | | |
| --- | --- | --- | --- | --- | --- | --- |
| ***Dog*** | ***1-1*** | ***1-2*** | ***1-3*** | ***2-1*** | ***2-2*** | ***2-3*** |
| 0 | BLQ | BLQ | BLQ | BLQ | BLQ | BLQ |
| 0.25 | 0.9615 | 1.2225 | 1.5468 | 1.4373 | 2.1189 | 1.858 |
| 0.5 | 1.3875 | 1.2452 | 1.7298 | 1.9245 | 2.1357 | 3.0445 |
| 0.75 | 1.4594 | 1.2048 | 1.8208 | 0.8551 | 2.291 | 2.5714 |
| 1 | 1.6091 | 1.2033 | 1.6462 | 1.7056 | 2.309 | 2.5612 |
| 1.5 | 0.3367 | 0.3982 | 0.2844 | 0.2145 | 0.3487 | 0.2271 |
| 2 | 0.1618 | 0.1694 | 0.0796 | 0.0762 | 0.1215 | 0.0558 |
| 4 | 0.0053 | 0.0052 | 0.0029 | 0.0036 | 0.0037 | 0.0026 |
| 6 | 0.002 | 0.0021 | 0.0012 | 0.0013 | BLQ | 0.0004 |
| 8 | 0.0015 | 0.0015 | 0.0008 | 0.0007 | BLQ | BLQ |
| 12 | 0.0007 | 0.0006 | 0.0004 | 0.0003 | BLQ | BLQ |
| 24 | 0.0005 | 0.0004 | BLQ | BLQ | 0.0038 | BLQ |
| 48 | 0.0005 | 0.0003 | BLQ | BLQ | BLQ | BLQ |
| 72 | 0.0005 |  | BLQ | 0.0028 | 0.0004 | BLQ |
| 144 | 0.0005 |  |  | BLQ | BLQ | BLQ |
| 168 |  | BLQ |  |  |  |  |
| 192 | 0.0005 | BLQ | BLQ | BLQ | BLQ | BLQ |
| 216 |  | BLQ |  |  |  |  |
| 240 | 0.0005 |  | BLQ | 0.0006 | BLQ | BLQ |
| 312 | BLQ | 0.0014 | BLQ | BLQ | BLQ | BLQ |
| 384 |  | 0.0003 |  |  |  |  |
| 408 | BLQ |  | BLQ | BLQ | BLQ | BLQ |
| 528 | BLQ | BLQ | BLQ | BLQ | BLQ | BLQ |
| 672 | BLQ | BLQ | BLQ | BLQ | 0.0004 | BLQ |

BLQ = below limit of quantification

**Table S6** – Isavuconazonium pharmacokinetic parameters for individual dogs administered intravenous isavuconazonium sulfate.

| **Parameter** | **Unit** | **Dog** | | | | | |
| --- | --- | --- | --- | --- | --- | --- | --- |
|  |  | ***1-1*** | ***1-2*** | ***1-3*** | ***2-1*** | ***2-2*** | ***2-3*** |
| Dose | mg/kg | 5.44 | 7.95 | 5.85 | 5.31 | 7.85 | 4.25 |
| λ_z_ | h^-1^ | 0.177 | 0.212 | 0.182 | 0.240 | 1.798 | 1.235 |
| t_1/2_ | h | 3.91 | 3.27 | 3.81 | 2.89 | 0.39 | 0.56 |
| T_max_ | h | 1.00 | 0.50 | 0.75 | 0.50 | 1.00 | 0.50 |
| C_max_ | μg/mL | 1.61 | 1.25 | 1.82 | 1.92 | 2.31 | 3.04 |
| AUC_0-t_ | μg*h/mL | 1.95 | 1.80 | 2.14 | 1.91 | 2.83 | 3.02 |
| AUC_0-∞_ | μg*h/mL | 1.95 | 1.80 | 2.15 | 1.91 | 2.83 | 3.02 |
| AUC_%extrap_ | % | 0.2 | 0.2 | 0.1 | 0.1 | 0.1 | 0.0 |
| V_z_ | L/kg | 15.7 | 20.8 | 15.0 | 11.6 | 1.5 | 1.1 |
| Cl | mL/kg/h | 2,789 | 4,408 | 2,725 | 2,780 | 2,770 | 1,408 |
| AUMC_0-t_ | μg*h^2^/mL | 1.89 | 1.75 | 1.75 | 1.55 | 2.27 | 2.27 |
| AUMC_0-∞_ | μg*h^2^/mL | 1.96 | 1.80 | 1.79 | 1.57 | 2.28 | 2.27 |
| AUMC_%extrap_ | % | 3.6 | 2.6 | 2.2 | 1.3 | 0.4 | 0.1 |
| MRT | h | 0.50 | 0.50 | 0.33 | 0.32 | 0.31 | 0.25 |
| V_ss_ | L/kg | 1.4 | 2.2 | 0.9 | 0.9 | 0.8 | 0.4 |

λ_z_ = terminal rate constant; t_1/2_ = terminal half-life; T_max_ = time at maximum concentration; C_max_ = maximum concentration; C_max_/D = maximum concentration normalized to dose; AUC_0-t_ = observed area under the curve; AUC_0-t_/D = observed area under the curve normalized to dose; AUC_0-∞_ = area under the curve extrapolated to infinity; AUC_0-∞_/D = area under the curve extrapolated to infinity normalized to dose; AUC_%extrap_ = percent area under the curve extrapolated; V_z_ = volume of distribution by the area method; Cl = clearance; AUMC_0-t_ = observed area under the moment curve; AUMC_0-∞_ = area under the moment curve extrapolated to infinity; AUMC_%extrap_ = percent area under the moment curve extrapolated; MRT = mean residence time; V_ss_ = volume of distribution at steady state.

**Table S7** – Isavuconazole concentrations for dogs administered isavuconazonium sulfate intravenously. Dogs 1-1, 1-2, and 1-3 received the intravenous formulation first, while dogs 2-1, 2-2, and 2-3 received the intravenous formulation following the washout period.

| **Time (h)** | **Isavuconazole (μg/mL)** | | | | | |
| --- | --- | --- | --- | --- | --- | --- |
| ***Dog*** | ***1-1*** | ***1-2*** | ***1-3*** | ***2-1*** | ***2-2*** | ***2-3*** |
| 0 | BLQ | BLQ | BLQ | BLQ | BLQ | BLQ |
| 0.25 | 0.9173 | 1.5259 | 1.5661 | 1.4605 | 1.3076 | 1.2542 |
| 0.5 | 1.5846 | 1.7893 | 1.3667 | 1.8076 | 1.8253 | 3.2486 |
| 0.75 | 1.6783 | 2.0081 | 1.6477 | 3.1687 | 2.3926 | 2.4095 |
| 1 | 2.0582 | 2.3860 | 2.2831 | 2.0675 | 2.6934 | 2.6654 |
| 1.5 | 0.7715 | 1.2934 | 0.5858 | 0.6398 | 1.2742 | 0.8922 |
| 2 | 0.7089 | 1.0488 | 0.3727 | 0.4710 | 0.9941 | 0.6908 |
| 4 | 0.4055 | 0.4642 | 0.1995 | 0.3011 | 0.4804 | 0.3643 |
| 6 | 0.3032 | 0.3361 | 0.1322 | 0.1882 | 0.3364 | 0.2697 |
| 8 | 0.2317 | 0.2285 | 0.0938 | 0.1698 | 0.2804 | 0.2109 |
| 12 | 0.1410 | 0.1398 | 0.0547 | 0.1097 | 0.1842 | 0.1482 |
| 24 | 0.0537 | 0.0631 | 0.0289 | 0.0671 | 0.0927 | 0.0677 |
| 48 | 0.0152 | 0.0285 | 0.0095 | 0.0202 | 0.0236 | 0.0265 |
| 72 | 0.0066 |  | 0.0055 | 0.0172 | 0.0104 | 0.0168 |
| 144 | 0.0015 |  |  | 0.0038 | 0.0038 | 0.0073 |
| 168 |  | 0.0037 |  |  |  |  |
| 192 | 0.0014 | 0.0031 | 0.0013 | 0.0038 | 0.0027 | 0.0017 |
| 216 |  | 0.0020 |  |  |  |  |
| 240 | BLQ |  | 0.0010 | 0.0016 | 0.0912 | 0.0017 |
| 312 | BLQ | 0.0012 | 0.0007 | 0.0009 | 0.0004 | 0.0016 |
| 384 |  | 0.0008 |  |  |  |  |
| 408 | BLQ |  | 0.0000 | 0.0006 | BLQ | 0.0008 |
| 528 | BLQ | 0.0007 | BLQ | BLQ | BLQ | 0.0004 |
| 672 | BLQ | BLQ | BLQ | BLQ | BLQ | BLQ |

**Table S8** – Isavuconazole pharmacokinetic parameters for individual dogs administered intravenous isavuconazonium sulfate.

| **Parameter** | **Unit** | **Dog** | | | | | |
| --- | --- | --- | --- | --- | --- | --- | --- |
|  |  | ***1-1*** | ***1-2*** | ***1-3*** | ***2-1*** | ***2-2*** | ***2-3*** |
| Dose | mg/kg | 2.92 | 4.27 | 3.14 | 2.86 | 4.22 | 2.28 |
| λ_z_ | h^-1^ | 0.017 | 0.005 | 0.005 | 0.006 | 0.013 | 0.006 |
| t_1/2_ | h | 41 | 147 | 135 | 121 | 52 | 108 |
| T_max_ | h | 1.00 | 1.00 | 1.00 | 0.75 | 1.00 | 0.50 |
| C_max_ | μg/mL | 2.06 | 2.39 | 2.28 | 3.17 | 2.69 | 3.25 |
| AUC_0-t_ | μg*h/mL | 8.10 | 11.47 | 5.48 | 8.79 | 11.43 | 10.65 |
| AUC_0-∞_ | μg*h/mL | 8.18 | 11.62 | 5.62 | 8.89 | 11.46 | 10.71 |
| AUC_%extrap_ | % | 1.0 | 1.3 | 2.4 | 1.2 | 0.3 | 0.6 |
| V_z_ | L/kg | 21.2 | 77.7 | 108.7 | 56.0 | 27.4 | 33.3 |
| Cl | mL/kg/h | 357 | 367 | 559 | 322 | 368 | 213 |
| AUMC_0-t_ | μg*h^2^/mL | 113 | 334 | 109 | 261 | 225 | 341 |
| AUMC_0-∞_ | μg*h^2^/mL | 134 | 443 | 178 | 321 | 236 | 384 |
| AUMC_%extrap_ | % | 15.7 | 24.7 | 38.8 | 18.9 | 4.9 | 11.2 |
| MRT | h | 16 | 38 | 31 | 36 | 20 | 35 |
| V_ss_ | L/kg | 5.6 | 13.8 | 17.4 | 11.5 | 7.4 | 7.5 |

λ_z_ = terminal rate constant; t_1/2_ = terminal half-life; T_max_ = time at maximum concentration; C_max_ = maximum concentration; C_max_/D = maximum concentration normalized to dose; AUC_0-t_ = observed area under the curve; AUC_0-t_/D = observed area under the curve normalized to dose; AUC_0-∞_ = area under the curve extrapolated to infinity; AUC_0-∞_/D = area under the curve extrapolated to infinity normalized to dose; AUC_%extrap_ = percent area under the curve extrapolated; V_z_ = volume of distribution by the area method; Cl = clearance; AUMC_0-t_ = observed area under the moment curve; AUMC_0-∞_ = area under the moment curve extrapolated to infinity; AUMC_%extrap_ = percent area under the moment curve extrapolated; MRT = mean residence time; V_ss_ = volume of distribution at steady state.

**Table S9** – Isavuconazonium concentrations for dogs administered isavuconazonium sulfate orally. Dogs 1-1, 1-2, and 1-3 received the oral formulation after the washout period, while dogs 2-1, 2-2, and 2-3 received the oral formulation first.

| **Time (h)** | **Isavuconazonium (μg/mL)** | | | | | |
| --- | --- | --- | --- | --- | --- | --- |
| ***Dog*** | ***1-1*** | ***1-2*** | ***1-3*** | ***2-1*** | ***2-2*** | ***2-3*** |
| 0 | BLQ | BLQ | BLQ | BLQ | BLQ | BLQ |
| 0.25 | BLQ | BLQ | BLQ | BLQ | BLQ | 0.0012 |
| 0.5 | BLQ | BLQ | BLQ | BLQ | BLQ | BLQ |
| 0.75 | BLQ | BLQ | BLQ | BLQ | 0.0062 | BLQ |
| 1 | BLQ | BLQ | BLQ | BLQ | BLQ | BLQ |
| 1.5 | BLQ | 0.0009 | BLQ | 0.0008 | BLQ | BLQ |
| 2 | 0.0004 | 0.0013 | BLQ | 0.0014 | 0.002 | 0.0024 |
| 4 | 0.0007 | 0.0007 | BLQ | 0.0003 | 0.0041 | 0.0008 |
| 6 | 0.0003 | BLQ | BLQ | BLQ | BLQ | BLQ |
| 8 | BLQ | BLQ | BLQ | BLQ | BLQ | BLQ |
| 12 | BLQ | BLQ | BLQ | BLQ | BLQ | BLQ |
| 24 | BLQ | BLQ | BLQ | BLQ | BLQ | BLQ |
| 48 | BLQ | BLQ | BLQ | BLQ | BLQ | BLQ |
| 72 | 0.0004 |  | BLQ | BLQ | BLQ | BLQ |
| 144 | 0.0008 | BLQ | BLQ | BLQ | BLQ | BLQ |
| 168 |  |  | BLQ |  |  |  |
| 192 | BLQ | BLQ |  | BLQ | BLQ | BLQ |
| 216 |  | BLQ |  |  |  |  |
| 240 | BLQ |  | BLQ | BLQ | 0.0016 |  |
| 312 | BLQ | BLQ | 0.0003 | BLQ | 0.002 | BLQ |
| 384 |  | BLQ |  |  |  |  |
| 408 | BLQ |  | BLQ | BLQ | BLQ | BLQ |
| 528 | BLQ | BLQ | BLQ | BLQ | BLQ | BLQ |
| 672 | BLQ | BLQ | BLQ | BLQ | BLQ |  |
| 744 |  |  |  |  |  | BLQ |

BLQ = below limit of quantification

**Table S10** – Isavuconazole concentrations for dogs administered isavuconazonium sulfate orally. Dogs 1-1, 1-2, and 1-3 received the oral formulation after the washout period, while dogs 2-1, 2-2, and 2-3 received the oral formulation first.

| **Time (h)** | **Isavuconazole (μg/mL)** | | | | | |
| --- | --- | --- | --- | --- | --- | --- |
| ***Dog*** | ***1-1*** | ***1-2*** | ***1-3*** | ***2-1*** | ***2-2*** | ***2-3*** |
| 0 | BLQ | BLQ | BLQ | BLQ | BLQ | BLQ |
| 0.25 | BLQ | BLQ | BLQ | BLQ | BLQ | BLQ |
| 0.5 | BLQ | BLQ | BLQ | BLQ | BLQ | BLQ |
| 0.75 | BLQ | BLQ | BLQ | BLQ | BLQ | BLQ |
| 1 | BLQ | BLQ | BLQ | 0.0014 | BLQ | BLQ |
| 1.5 | 0.0071 | 0.0011 | BLQ | 0.0146 | BLQ | BLQ |
| 2 | 0.0076 | 0.0018 | BLQ | 0.0509 | 0.0077 | 0.0013 |
| 4 | 0.0303 | 0.4211 | 0.005 | 0.3367 | 0.1904 | 0.0086 |
| 6 | 0.7468 | 0.8696 | 0.458 | 0.3695 | 1.0045 | 0.0214 |
| 8 | 0.6072 | 0.5358 | 0.3576 | 0.2846 | 0.8238 | 0.0409 |
| 12 | 0.3047 | 0.2096 | 0.0881 | 0.1518 | 0.3829 | 0.4135 |
| 24 | 0.0751 | 0.0527 | 0.0261 | 0.0914 | 0.1021 | 0.1096 |
| 48 | 0.0154 | 0.0197 | 0.0079 | 0.029 | 0.0251 | 0.0326 |
| 72 | 0.0049 |  | 0.004 | 0.01 | 0.0087 | 0.0142 |
| 144 | 0.0013 | 0.0035 | 0.0015 | 0.0033 | 0.0023 | 0.0056 |
| 168 |  |  | 0.0012 |  |  |  |
| 192 | 0.0011 | 0.0021 |  | 0.0021 | 0.0015 | 0.0036 |
| 216 |  | 0.0017 |  |  |  |  |
| 240 | 0.0007 |  | 0.0009 | 0.0014 | 0.0011 |  |
| 312 | BLQ | 0.0008 | 0.0005 | 0.001 | 0.0008 | 0.0012 |
| 384 |  | 0.0007 |  |  |  |  |
| 408 | BLQ |  | BLQ | 0.0008 | BLQ | 0.0007 |
| 528 | BLQ | BLQ | BLQ | 0.0006 | BLQ | 0.0004 |
| 672 | BLQ | BLQ | BLQ | BLQ | BLQ |  |
| 744 |  |  |  |  |  | BLQ |

BLQ = below limit of quantification

**Table S11** – Isavuconazole pharmacokinetic parameters for individual dogs administered oral isavuconazonium sulfate.

| **Parameter** | **Units** | **Dog** | | | | | |
| --- | --- | --- | --- | --- | --- | --- | --- |
|  |  | ***1-1*** | ***1-2*** | ***1-3*** | ***2-1*** | ***2-2*** | ***2-3*** |
| Dose | mg/kg | 2.92 | 4.27 | 3.14 | 2.86 | 4.22 | 2.28 |
| λ_z_ | h^-1^ | 0.011 | 0.007 | 0.006 | 0.002 | 0.005 | 0.005 |
| t_1/2_ | h | 62 | 102 | 112 | 293 | 134 | 137 |
| T_max_ | h | 6.00 | 6.00 | 6.00 | 6.00 | 6.00 | 12.00 |
| C_max_ | μg/mL | 0.75 | 0.87 | 0.46 | 0.37 | 1.00 | 0.41 |
| AUC_0-t_ | μg*h/mL | 7.93 | 8.52 | 3.77 | 6.96 | 11.10 | 7.80 |
| AUC_0-∞_ | μg*h/mL | 7.99 | 8.62 | 3.85 | 7.22 | 11.25 | 7.88 |
| AUC_%extrap_ | % | 0.8 | 1.2 | 2.1 | 3.5 | 1.4 | 1.0 |
| AUMC_0-t_ | μg*h^2^/mL | 146 | 230 | 98 | 285 | 232 | 365 |
| AUMC_0-∞_ | μg*h^2^/mL | 166 | 285 | 136 | 526 | 310 | 423 |
| AUMC_%extrap_ | % | 12.4 | 19.3 | 28.1 | 45.9 | 25.2 | 13.5 |
| MRT | h | 21 | 33 | 35 | 73 | 28 | 54 |
| F | % | 97.7 | 74.2 | 68.5 | 81.2 | 98.2 | 73.5 |

λ_z_ = terminal rate constant; t_1/2_ = terminal half-life; T_max_ = time at maximum concentration; C_max_ = maximum concentration; C_max_/D = maximum concentration normalized to dose; AUC_0-t_ = observed area under the curve; AUC_0-t_/D = observed area under the curve normalized to dose; AUC_0-∞_ = area under the curve extrapolated to infinity; AUC_0-∞_/D = area under the curve extrapolated to infinity normalized to dose; AUC_%extrap_ = percent area under the curve extrapolated; AUMC_0-t_ = observed area under the moment curve; AUMC_0-∞_ = area under the moment curve extrapolated to infinity; AUMC_%extrap_ = percent area under the moment curve extrapolated; MRT = mean residence time; F = bioavailability.
